# Melanoma Cancer Stem Cells: Markers and Functions

**DOI:** 10.3390/cancers8030034

**Published:** 2016-03-11

**Authors:** Giorgio Parmiani

**Affiliations:** Italian Network for Bioimmunotherapy of Tumors, Division of Medical Oncology and Immunotherapy, University Hospital, Viale Bracci, 16, 53100 Siena, Italy; giorgio.parmiani@fastwebnet.it; Tel.: +39-333-687-889

**Keywords:** human melanoma, cancer stem cells, biomarkers, immune checkpoint antibodies, immunosuppression

## Abstract

The discovery of cancer stem cells (CSCs) in human solid tumors has allowed a better understanding of the biology and neoplastic transformation of normal melanocytes, and the possible mechanisms by which melanoma cells acquire tumorigenicity. In this review I summarize the literature findings on the potential biomarkers of melanoma CSCs, their presence in the melanoma cell populations, the interaction with the immune system (with both T and NK cells) and the role of melanoma CSCs in the clinics. Given the extraordinary progress in the therapy of melanoma caused by immune checkpoint antibodies blockade, I discuss how these antibodies can work by the activation of melanoma infiltrating T cells specifically recognizing neo-antigens expressed even by melanoma CSCs. This is the mechanism that can induce a regression of the metastatic melanomas.

## 1. Melanoma CSCs: Their Frequency as Potential Markers in the Melanoma Microenvironment

Between 1995–2010 many studies reported the presence of a particular subpopulation of cancer cells characterized by self-renewability and the capacity to initiate, replenish and expand human tumors. These cancer cells with stem cells features and defined as cancer stem cells (CSCs) or tumor-initiating cells have been isolated from different human solid tumors, including melanoma. CSCs have been reported to express a variety of markers (e.g., CD34, ALDH1, CD271, CD44, ALDH1, JARID1) none of which, however, has been shown to be truly CSC-specific [1,2,3,4,5].

This theory implies that therapeutic destruction of CSCs should impair tumor growth [6]. In addition, one should consider the possibility that different subpopulations of CSCs may exist within single tumors (intratumoral heterogeneity) including melanoma [7,8,9] and/or among different tumors (intertumoral heterogeneity). In the absence of reliable markers that may define such subpopulations it will be difficult, if not impossible, to manipulate/eliminate tumorigenic CSCs and to use them as a target for therapeutics.

A relevant problem of the theory of CSCs lies in the frequency and instability of CSCs in each human solid tumor that in the majority of cases studied have been reported to be quite low, thus questioning the potential pro-tumorigenic functions of these cells. A notable exception is melanoma where the frequency and growth of single human melanoma CSCs as assessed by xenotransplantation in immunodeficient gene modified mice, could reach 27% [10].

### Markers

Several potential bio-markers of CSCs have been described as expressed by human solid tumors including melanoma. [11]. A potential list of these markers includes the following:

Neural crest nerve growth factor receptor CD271 with an estimated frequency of 6.4%–75% [12,13]; CD271+ melanoma cells are tumorigenic in immunodeficient (NOD/SCID) mice.

Nanog, Oct3/4 transcription factors were found to be more expressed in melanosphere than in adherent melanoma cell lines [8].

CD20, a cell surface marker of normal B cells was reported to be increased in melanoma [14] particularly in melanoma cells growing as multispheres [11,14].

CD133 [15] is a five transmembrane domain glycoprotein used to isolate CSCs from different types of tumors. CD133 is a melanoma immunogenic target [15] whose expression is often associated with expression of cancer/testis antigens [16,17].

Signalling pathways of normal stem cells like those involving Wnt, Notch. HedgeHog can be activated in melanoma CSCs [1,2,3,4,5].

ALDH1 (aldehyde dehydrogenase) is a potential marker of CSCs associated with multidrug and immunological resistance in different types of human solid tumors [15,16,17,18,19,20,21].

ABCB1, ABCB5, ABCG2: melanocytes and melanoma cells were found to variably express some of these markers (particularly ABCB5) but they were more frequently expressed in melanospheres and cell lines [8].

Sox10: It is of interest that melanoma CSC can grow *in vitro* in the presence of embryonic stem cell medium as non-adherent tumorigenic spheres while non-CSCs of the same lesion can grow only as adherent cell cultures [3,6]. In addition, melanoma appears to be the tumor type with the highest frequency of CSCs (up to 27%), while other neoplasms show a very limited presence of these cells (e.g., 0.0001%) [8].

## 2. *In Vitro* and *in Vivo* Functions of CSCs (Immunosuppression)

To be effective in their tumor-promoting activity, CSCs need to escape the patients’ anti-tumor immune-mediated reactions. Like CSCs of other neoplasms, melanoma CSCs have been shown to express a variety of antigens (including differentiation and cancer testis antigens) known to be recognized by T cells (e.g., MelanA/Mart1, HMB45, tyrosinase, gp100, NYESO1) [18,19]. However, differentiation and cancer testis antigens usually elicit a weak response even in deliberately immunized melanoma patients which only rarely is associated with a clinical response [18,20,22].

To explain this, our group and that of other investigators demonstrated that CSCs from melanoma and from other human solid tumors can activate several mechanisms that allow them to survive in a hostile micro-environment and escape the patient’s immune reactions as it may occur with other tumors like glioblastoma and colorectal cancer [23,24].

Moreover, human tumors CSCs have been shown to be recognized and destroyed by autologous NK cells according to the differential expression of some markers (e.g., CD133, CD117, CD271) [23,24,25,26].

## 3. Chemoresistance of CSCs

Melanoma is known to be a tumor resistant to chemotherapy but it is not clear which are the mechanisms of such a resistance and whether they are different from those used by non-CSCs. However, studies on chemotherapy (paclitaxel and epirubicin-resistant breast CSCs) revealed that ALDH1 production and membrane-bound IL-4 was instrumental in defining chemotherapy-resistant breast CSCs [23,24,25,26,27].

## 4. Clinical Aspects

In addition, the recent discovery that tumorigenic cells, particularly melanoma cells expressing ABCB5+, can also express immune co-stimulatory and/or co-inhibitory molecules (e.g., PD-1/PD-L1; CTLA4) [14,20] has allowed an entirely new and effective therapeutic approach for metastatic melanoma patients and, more recently, even for other human solid tumors as well, based on the administration of immune checkpoint antibodies [26,27,28,29,30,31,32,33,34,35] that can trigger a strong, clinically effective T cell mediated anti-tumor response. This effect may be also due to the targeting of neo-antigens expressed by CSCs.

In fact, in a recent study we have determined the profile of mutated genes of human colorectal carcinoma cells (CRC) and of CSCs derived from tissue samples and found that identical mutated neo-antigens were expressed both in tissues and in cell lines deriving from such samples. In addition, we also showed that patients’ T cells could recognize the neo-antigens, particularly by those T cells deriving from tumor infiltrating lymphocytes (TILs) or lymph nodes enriched by T cells [24,25,27,28].

## 5. Conclusions

The definition of CSC markers in human melanoma is a crucial issue that will certainly be solved within a short time from now. This will allow a better definition of the functions of CSCs *in vivo*, their role in tumor formation and diffusion and the resistance to cellular immune response that patients can mount, upon checkpoint antibody administration, against immunogenic neo-antigens. As discussed above, CSCs may express the same or different neo-antigens of non-SCs revealed by appropriate genetic analysis that allows to define the mutation landscape of each melanoma. Thus it should be kept in mind that one or more neo-antigens may be necessary to trigger a strong cytotoxic effect against melanoma CSCs after injection of the checkpoint antibodies.
